# Predictive models of the risk of hospital admission and re-admission: current and future development

**DOI:** 10.1186/1472-6963-15-S2-A9

**Published:** 2015-06-15

**Authors:** Stephen Sutch, Klaus Lemke, Chad Abrams

**Affiliations:** 1 Johns Hopkins University, Baltimore, Maryland, 21218, USA

## Background

A number of models are available in the United States (US) and the United Kingdom (UK) for use in predicting the risk of hospitalization, from general and insured populations. These models are being used in order to respond to health policies, such as Pay for Performance measures, aimed at reducing unnecessary hospital admissions, and to help patients avoid hospital admissions that are expensive and create risks to patient safety. These predictive models are being used for a variety of purposes including: screening patients for Case Management Programs and/or Disease Management Programs, organizational profiling, and assessing financial risk.

## Materials and methods

The analysis was conducted using data from US commercial and Medicare populations, and from the UK National Health Service General Populations. The predictive models were derived using patient-level data (including population health and primary, community and secondary care data), with the classification of diagnostic, pharmaceutical, and historic utilization data. The Johns Hopkins Adjusted Clinical Groups (ACG) Case-Mix System was used in order to reduce the number of variables and provide measures of multi-morbidity.

Logistic Regression was undertaken to produce models on the dichotomous outcomes of hospitalization within 6-12 months, emergency and/or unplanned hospitalization within 12 months, re-hospitalization within 30 days, and long-stay hospitalization.

The models were validated using split-half method, and providing ROC (Receiver Operating Characteristic) analyses to compare different model performance.

## Results

The models work well in explaining the top 1% and 5% of data; they also perform well in discriminating risk occurring “lower in the population pyramid” in order to identify potential emerging risk. The results demonstrate the importance of case-mix classifications to reduce data complexity and provide robust measures of multi-morbidity.

## Conclusions

While these models focus on identifying the highest-risk individuals, there is also a public health interest in recognizing earlier and emerging risks where more preventative methods can be informed, such as chronic disease self-management programs. In their current form, these models are being used to identify populations, but work on newly evolving data from Electronic Health Records, Personal Health Records, and Social Care data is expected to provide greater insight into both these populations and those with highest need.

